# A WEB-BASED MOBILE APPLICATION TO SUPPORT SOCIAL ENGAGEMENT IN PERSONS WITH DEMENTIA

**DOI:** 10.1093/geroni/igz038.1738

**Published:** 2019-11-08

**Authors:** Hayley R McCarron, Rachel Zmora, Joseph E Gaugler

**Affiliations:** 1 University of Minnesota School of Public Health, Minneapolis, Minnesota, United States; 2 University of Minnesota, Minneapolis, Minnesota, United States; 3 University of Minnesota - School of Public Health, Division of Health Policy and Management, Minneapolis, Minnesota, United States

## Abstract

Assistive technology has been recognized as a promising tool to improve the lives of persons living with dementia and their caregivers. The use of assistive technology in dementia care is expanding, although it is most often applied to manage care and promote safety. There is a lack of assistive technology designed to aid persons with dementia to participate in meaningful activities. The present study utilizes a pilot randomized controlled trial (RCT) design to evaluate the feasibility, acceptability, and effects of an assistive technology device, the Social Support Aid (SSA), designed to assist persons with dementia engage in social interaction. Quantitative data were collected initially and three and six months later, and participants in the technology group participated in qualitative interviews. Feasibility and utility scores indicated that participants felt neutral about the technology. Use of the SSA was not significantly associated with changes in quality of social interactions or quality of life measures over the six months of follow-up (P > .05). The qualitative analysis revealed three themes that described how and why the SSA worked or did not: (1) outcomes (2) reasons why (not) useful and (3) recommendations. There is a need to develop effective assistive technology that improves the quality of life of persons with dementia. Assistive technology that allows persons living with dementia to maintain some level of autonomy should be a priority for future research. Results provide recommendations for future assistive technology development and evaluation.

